# Bas-relief modeling from RGB monocular images with regional division characteristics

**DOI:** 10.1038/s41598-022-24974-0

**Published:** 2022-12-15

**Authors:** Xinli Wu, Yun Zhao, Jiali Luo, Minxiong Zhang, Wenzhen Yang

**Affiliations:** 1 Zhejiang Sci-Tech University, Hangzhou, 310018 China; 2 Research Center for Humanoid Sensing, Intelligent Perception Research Institute of Zhejiang Lab, Hangzhou, 311121 China

**Keywords:** Computational science, Computer science, Information technology

## Abstract

Traditional Bas-relief modeling methods are often limited to inefficient and difficult to be altered after the product is formed. This paper presents a novel method for bas-relief modeling from RGB monocular images with regional division characteristics. The problem discussed in this paper involves edge detection, region division, height value recovery and three-dimensional reconstruction of image. In our framework, we can automatically obtain the pixel height of each area in the image and can adjust the concave–convex relationship of each image area to obtain a bas-relief modeling which can be printed directly in 3D. The edge detection algorithm used Gaussian difference pyramid to combine the luminance information and chrominance information of digital image; the regions of the RGB monocular image are divided by the improved connected-component labeling algorithm;and the 3D pixel point cloud of each region is calculated by the shape-from-shading algorithm. Different from previous work, our method can fully obtain the image height field data and completely restored the depth information,which makes it possible to use any RGB monocular image for bas-relief modeling. Experiments with groups of images show that our method can effectively generate bas-relief modeling.

## Introduction

Bas-relief, a form of sculpture art representation, has the general characteristics of sculpture art and satisfies people’s visual and tactile feelings by fully leveraging the advantages of painting art in composition, subject matter, and spatial processing. There are two main types of reliefs: high relief and bas-relief (also called low relief). High relief is a type of sculpture where the figure stands out further from the ground with the most prominent elements of the composition being undercut, while bas-relief has a shall-lower overall depth in comparison with high relief. This paper focused on bas-relief.

Sculpture is a ubiquitous art form in human life. A sculptor gradually transforms a two-dimensional planar drawing to a three-dimensional (3D) representation by applying the shadow expression method. By using various materials to realize the 3D representation of different images, the history of relief sculpture has been promoted. Bas-relief, which is a form of sculpture art, not only has the general characteristics of sculpture art but also satisfies people’s visual and tactile feelings. It can fully leverage the advantages of painting art in composition, subject, matter, and space processing.

In this paper, we presents a novel method for bas-relief modeling from RGB monocular images with regional division characteristics. Our method can automatically obtain the pixel height of each area in the image and can adjust the concave-convex relationship of each image area to obtain a bas-relief model based on the RGB monocular image. First, the edge contour of an RGB monocular image is extracted and refined by the Gauss difference algorithm based on tangential flow. Subsequently, the complete image contour information is extracted and the region-based image segmentation is used to calibrate the region. This method has improved running speed and stability compared with the traditional algorithm. Second, the regions of the RGB monocular image are divided by the improved connected-component labeling algorithm. In the traditional region calibration algorithm, the contour search strategy and the inner and outer contour definition rules of the image considered result in a low region division efficiency. This study uses an improved contour-based calibration algorithm. Then, the 3D pixel point cloud of each region is calculated by the shape-from-shading algorithm. The concave–convex relationships among these regions can be adjusted by human–computer interaction to form a reasonable bas-relief model. Lastly, the bas-relief model is obtained through triangular reconstruction using the Delaunay triangulation algorithm. The final bas-relief modeling effect is displayed by OpenGL.

*Contribution* In this study, six groups of images are selected for conducting regional division tests, and the results obtained by the proposed method and other existing methods are compared. The proposed algorithm shows improved image processing running time for different complexity levels compared with the traditional two-pass scanning method and seed filling method (by approximately 2 s) and with the contour tracking method (by approximately 4 s). Next, image depth recovery experiments are conducted on four sets of images, namely the “ treasure seal”, “Wen Emperor seal”, “lily pattern”, and “peacock pattern”, and the results are compared. The depth of the image obtained by the traditional algorithm is generally lower than the actual plane, and the relative height of each region is not consistent with the actual situation. The proposed algorithm provides height values consistent with the height value information judged in the original image and adjusts the accurate concave–convex relationships. Moreover, the noise in the image is reduced and relatively smooth surfaces are obtained, with accurate concave–convex relationships. The proposed bas-relief model based on RGB monocular images can automatically determine the pixel height of each image area in the image and adjust the concave–convex relationship of each image area. In addition, it can recover the 3D model of the object from the image, enrich the object of bas-relief modeling, and expand the creation space of bas-relief, thereby improving the production efficiency of the bas-relief model based on RGB monocular images. The method has certain shortcomings, which require further exploration. For example, during the process of image contour extraction for region division, small differences exist between the obtained result and the actual situation, which can in turn affect the image depth recovery in the later stage. In addition, partial distortion may occur in the process of 3D reconstruction, which requires further research on point cloud data processing to reconstruct a high-quality three-dimensional surface.

## Related work

In recent years, scholars around the world have conducted in-depth research on bas-relief modeling. The use of images for bas-relief modeling is usually divided into three categories: bas-relief modeling based on 3D models, and bas-relief modeling based on two-dimensional images.

The bas-relief modeling method based on the 3D model compression method mainly involves scanning of the target object from multiple angles using a scanning device to obtain a 3D model of the object in the real world and finally reconstructing a complete 3D shape. Cignoni^1^, who is the pioneer in the research on bas-relief production through 3D model mapping, applied perspective projection and depth compression to generate relief models. This laid the foundation for subsequent research on 3D model mapping. Later, Weyrich et al.^2^ used a nonlinear compression function to compress the gradient domain. This method preserves the details of the image well and provides gentler contours. Zhang et al.^3^ directly compressed the input 3D model to obtain a suitable depth of the dynamic range. However, the high dynamic range (HDR)-based method can omit some details in the model, resulting in the bas-relief model missing the details in a small area. Zhang et al.^4^ proposed a relief that adaptively generates lighting conditions for the lighting effect on the appearance of bas-relief. Wei et al.^5^ used a two-step mesh smoothing mechanism as a bridge to perform appropriate operations on the smooth base layer and the detail layer, which can retain more details during compression. In the bas-relief production process of 3D model mapping, the reverse operation from the gradient domain to the spatial domain is relatively time consuming. Kerber et al.^6^ used programmable hardware to realize the real-time generation of relief models. Zhang et al.^7^ proposed a real-time interactive relief texture model construction method through gradient domain compression and texture mapping.

The bas-relief modeling based on two-dimensional images. Compared with 3D models, 2D images are easier to obtain, which expanding the application range of digital bas-relief. But the difficulty is that the color, brightness, texture and other geometric information of the object reflected in the image is not sufficient and can not be very good express. In order to overcome this problem, in recent years, many researchers have started from two-dimensional images. The key to the bas-relief modeling method based on two-dimensional image depth estimation method is to obtain the depth information of the image, currently, image depth information can be obtained through two main methods. One is to use a depth camera to obtain object depth information. A depth image contains pixels, each of which stores the depth value of a specific point of the target object. In the case that the internal and external parameters of the camera are known, the complete depth information of the object to be obtained is acquired from the photographs taken from multiple angles. Finally, the complete depth information of the target object is obtained through stitching. The other method is to recover the depth information of the pixels through the image, namely using multi-angle depth image stitching. Horn^8^ first proposed the modeling method of SFS 3D model in the 1980s. The idea is to calculate the lighting angle based on the shadow light and shade information of the digital image for 3D modeling. Pentland^9^ proposed a local analysis method, which considers that each pixel has the same image intensity and the first and second derivatives of a point on the Lambert surface with the same principal curvature, so as to recover the 3D height information of the image. Alexa and Matusik^10^ proposed a discrete model on the relief surface, and introduced the necessary degrees of freedom to overcome the theoretical limitations and practical requirements of the shadow on the shape. The iterative least square optimization method was used to determine the discrete surface. However, the model results were greatly affected by the changes in light and were prone to distortion. Wang Song et al.^11^ use the object area detection and slope function to process the low frequency components in the image, and the interactive method is used to process the high frequency components in the image. After the two parts are processed separately, the normalized grayscale images of the two parts are finally superimposed to obtain the three-dimensional relief height. Xia Meng^12^ divided the relief model into three parts: low frequency, medium frequency and high frequency, and added the gray control chart of the object area and the region boundary seed constraint information to obtain a complete relief model. Sohn^13^ realized a bas-relief generation system on mobile phones and computers by taking a group of ordinary photos as input and reflecting scene details and compressed information based on image pixel values.

In addition, some studies mainly use the method of line and region division. Kolomenkin^14^ proposed a method to automatically reconstruct relief from line painting, so as to solve the four problems of sparsity of lines, fuzziness of line painting, a large number of strokes in line painting, and interaction between closed curves. Zeng^15^ applied region extraction and depth relationship inference to the process of generating relief from a single image. This method outlines the contour and chisels out the layers with the process of manually making relief, so as to obtain the relief with correct global shape. The relatively simple image test results in the experiment are better. Wei et al.^16^ made two kinds of bas-relief. One is to retain the details of bas-relief, which can maintain the overall shape and details of 3D models. The other is the shallow relief modeling with the structure reserved, which only retains the overall shape and ignores the details. It is intuitive to generate bas-relief from natural images and photos. However, sometimes the color, brightness and texture in the image cannot reflect the geometric properties of objects well, especially for objects with complex materials. Wu^17^ and To^18^ focus on extracting bas-relief modeling from face photos to form a face bas-relief model with correct overall geometric properties and stable appearance. Ji et al.^19^ presents a novel method for digital bas-relief modeling with intuitive style control. He discussed the problem involves generating a discontinuity-free depth field with high compression of depth data while preserving or even enhancing fine details, which can be used to freely design bas-relief in normal image space instead of in object space, which makes it possible to use any popular image editing tools for bas-relief modeling. Zhang et al.^20^ focused on the Chinese calligraphy relief model, converted the calligraphy font into a triangle network, constructed a uniform height field by estimating the grid normal in the network, and converted the 2D font into a 2.5D shallow relief model. The uniform and non-uniform height fields are combined to facilitate shape operation, and provide users with a flexible way to interactively adjust the structure and details of the bas- relief model.

In summary, the current prerequisite for bas-relief modeling based on the 3D model through mapping is that the 3D model of the object already exists. However, most objects that are targeted for bas-relief representation do not have the original 3D models. Moreover, the complexity of the scene causes a low grid drawing efficiency after the point cloud is merged in the bas-relief modeling methods based on the image for depth recovery and those based on multi-images. Therefore, the merged network model needs to be simplified. Furthermore, the external equipment used by this method requires a high shooting location. Hence, this paper proposes a bas-relief modeling method for RGB monocular images based on regional division. Taking monocular images as the research object, this paper focuses on exploring the key technologies of image regional division, depth recovery, and surface 3D reconstruction. The proposed method can automatically obtain the pixel height value of each image area in the image and adjust the concave–convex relationship of each image area to obtain a bas-relief model based on RGB monocular images.

The 3D model method requires the 3D model of the object in advance. Bas-relief modeling based on the image depth restoration method usually either uses a depth camera to obtain object depth information or restores the depth information of pixels through the image. The complexity of the scene often results in a low grid drawing efficiency after the point cloud is merged. Therefore, the merged network model needs to be simplified. Moreover, this method uses an external device, which requires a high position for shooting. Bas-relief modeling based on the multi-image requires a short running time and has high efficiency in processing high resolution level images. This method requires compact external devices for acquiring the depth information and a low shooting position; however, it uses a relative more complex algorithm. Therefore, this study proposes a bas-relief modeling method based on region division. Monocular images are taken as the research object to explore the key techniques of region division, depth restoration, and surface 3D reconstruction. This method can automatically obtain the pixel height of each area in the image and can adjust the concave–convex relationship of each image area to obtain a bas-relief model based on the RGB monocular image.

## Bas-relief modeling based on a monocular image

**Figure 1:**
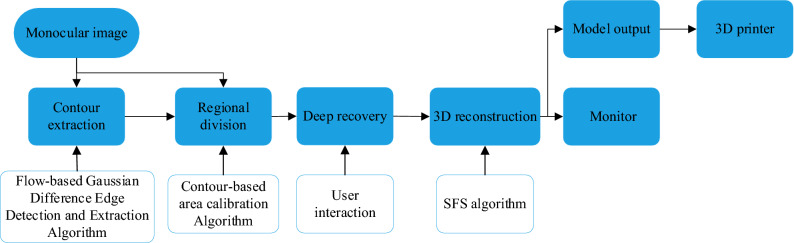
Main structure diagram.

First, the contour of an image is obtained from the monocular image, and a reasonable regional division is performed on the basis of the contour image extraction. The entire image is divided into multiple regions, each of which can be adjusted separately. On this basis, the initial depth of the image is obtained by the shape-from-shading (SFS) algorithm. Then, the relative heights between the regions are adjusted manually. Finally, the image depth information that is consistent with the actual situation is obtained. Through the triangulation algorithm, a patch reconstruction is performed on the recovered point cloud data, which finally results in a bas-relief model of the monocular image. The overall structure diagram is shown in Fig. 1.

### Regional division method based on image contour extraction

In this paper, the chroma information and brightness information of the image are combined. The flow-based Gaussian difference algorithm is adopted to extract the contour of the image. Finally, relatively complete image contour information is extracted. Furthermore, the contour-based image regional division is used for regional calibration. In view of the shortcomings of traditional algorithms, improved methods are proposed to enhance the running speed and stability of the algorithm.

**Figure 2:**
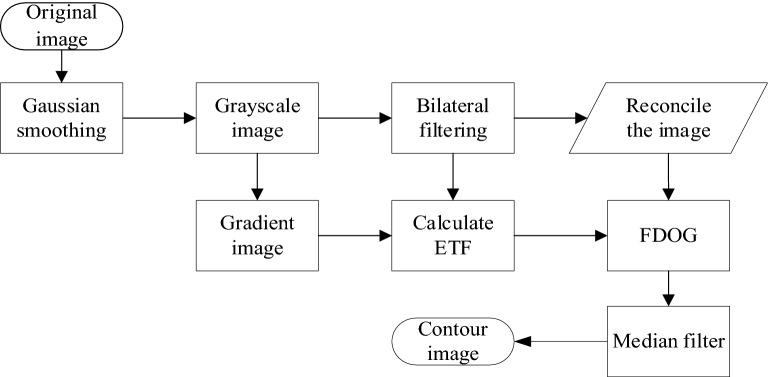
Contour extraction flow chart.

In general, the gray value of the target pixel in the digital image and that of the background pixel are quite different in the local range, although they still overlap in the global range. To save the extracted image contours perfectly at the edge features, this paper uses the algorithm proposed by Kang et al.^21^ to extract the image contours. The algorithm flow chart is shown in Fig. 2. After the original image is pre-processed, the flow-based Gaussian difference algorithm is used to extract the contour lines, which are then processed by a filtering method.

**Figure 3:**
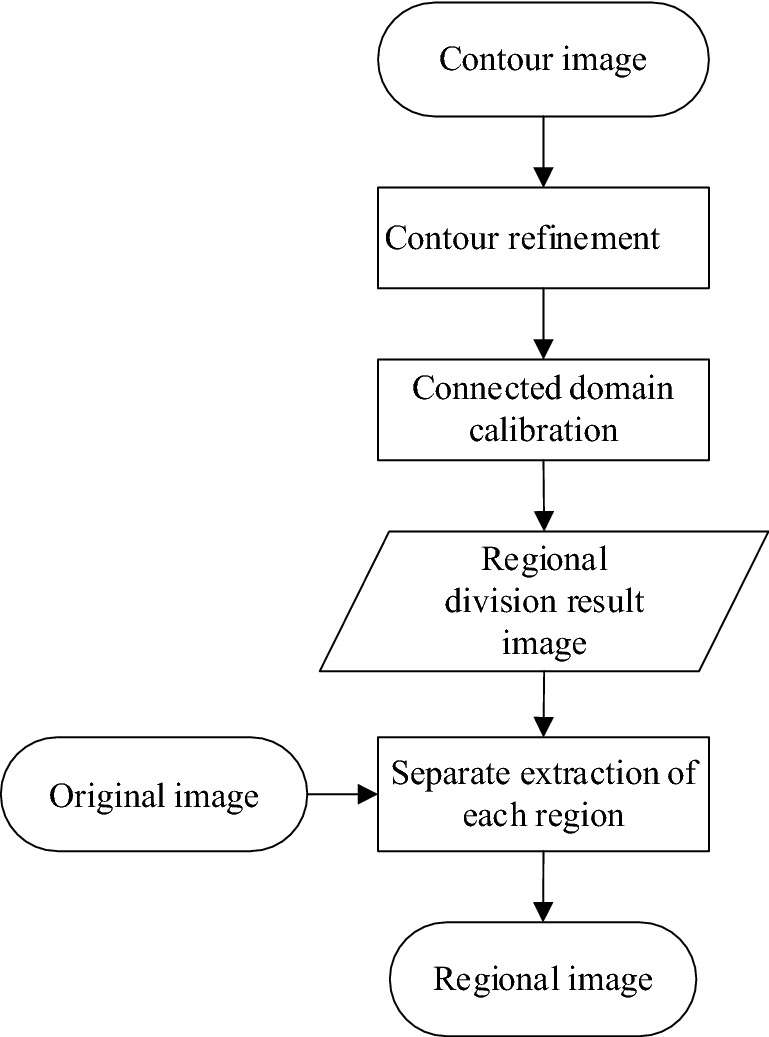
Contour-based regional division of an image.

In this paper, the region is divided on the basis of the contour image. In the traditional contour region calibration algorithm, the regional division efficiency is low because of the use of the contour search strategy and the internal and external contour definition rules of the image, and it is time consuming. Therefore, this paper improves the contour-based calibration algorithm. For this purpose, first, the extracted contour is refined, and the contour lines are refined into single pixels. Then, the improved regional calibration method is used to divide the image region. Figure 3 shows the improved algorithm flow chart.

**Figure 4:**
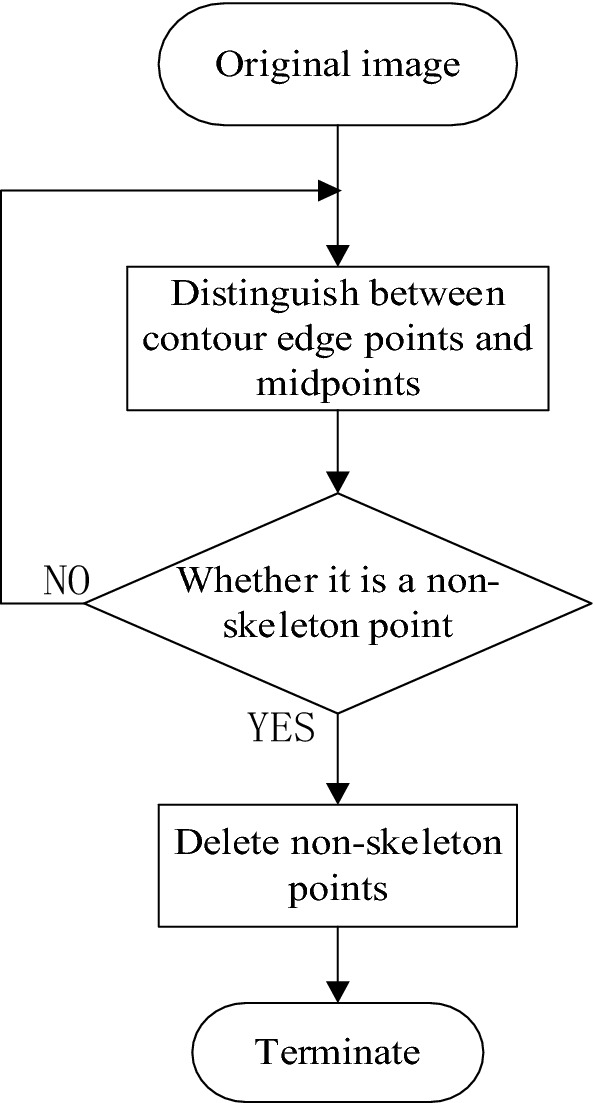
Contour image refinement flow chart.

Contour image refinement aims to remove unnecessary contour points and only retain the skeleton points of the contour. The specific process of contour image refinement is shown in Fig. 4.

In this paper, the contour marking algorithm is used to divide the region. Assume a binary image whose background is white and contour to be refined is black, the value of white pixels is 0, and that of black pixels is 1. The specific process is as follows.

1. From left to right, the image pixels are traversed from top to bottom. When the foreground pixel point $P$ is scanned (the first foreground pixel point to be traversed), the pixel point is not marked. At this time, this foreground pixel point is assigned with a new label number. Moreover, the contour of the pixel is traversed according to the connection rule, and it is assigned the same label number. Finally, the process returns to the original foreground pixel.
2. During the traversal process, if the traversed foreground pixel $P^{\prime}$ is encountered, traversal is continued to the right and the pixel is labeled with a value consistent with the label number of $P^{\prime}$.
3. In the traversal process, the contour is traversed when unlabeled foreground pixel $Q$ is encountered. All foreground pixels on the path are assigned new label numbers label+.
4. When the second labeled contour is encountered for the first time in the traversal process, the background pixel on the right is marked as the label number of the second contour. When the second labeled contour is encountered for the second time, the label number on the right is the same as the label number of the first contour.
5. The process terminates.

### Deep recovery

**Figure 5:**
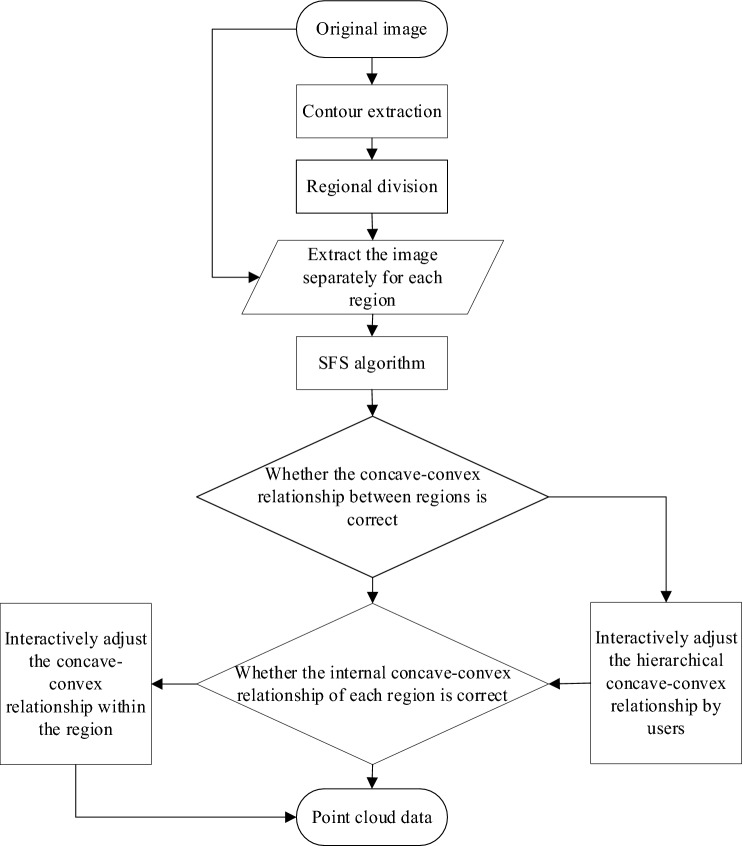
Flow chart of region-based image depth recovery.

The original point cloud data of the image pixels is obtained by using the SFS algorithm^12^. However, the difficulty of the SFS algorithm for image 3D surface reconstruction is that the relative concave–convex relationship between objects is blurred, owing to which the relative concave–convex relationship between image regions cannot be accurately judged^13^. Therefore, although this method can obtain satisfactory partial reconstruction effects, due its shortcomings, the overall structure of an image cannot be accurately achieved^14^. This paper proposes an interactive SFS algorithm to improve the 3D surface reconstruction effect of the traditional SFS algorithm. The specific process of the interactive algorithm is shown in Fig. 5.

The algorithm has low complexity and high running speed in the process of obtaining the solution. The specific solution process is as follows.

$$\begin{aligned} f[z(x,y)] =\, & E(x,y) - R(p(x,y),q(x,y)) \\ = \,& E(x,y) - \frac{{pp_{s} + qq_{s} + 1}}{{\sqrt {p_{{}}^{2} + q_{{}}^{2} + 1} \sqrt {p_{s}^{2} + q_{s}^{2} + 1} }} \\ =\, & 0 \\ \end{aligned}$$

where $E(x,y)$ is the image brightness; $R(x,y)$ is the image brightness reconstructed from the reflection model; $\left( { - p, - q,1} \right)^{T}$ is the normal vector of surface region; and $( - p_{s} , - q_{s} ,1)^{T}$ is the incident direction.

First, the reflection function $R(p,q)$ is discretized as follows.

$$\left\{ {\begin{array}{*{20}c} {p = \frac{\partial z}{{\partial x}} = z(x,y) - z(x - 1,y)} \\ {q = \frac{\partial z}{{\partial y}} = z(x,y) - z(x,y - 1)} \\ \end{array} } \right.$$

The brightness equation is

$$\begin{aligned} f[z(x,y)] = \,& E(x,y) - R(p(x,y),q(x,y)) \\ =\, & E(x,y) - R[z(x,y) - z(x - 1,y),z(x,y) - z(x,y - 1)] \\ =\, & 0 \\ \end{aligned}$$

Then, the Taylor formula is applied for expansion, taking low-order functions as the main processing object:

$$f[z(x,y)] \approx f[z^{n - 1} (x,y)] + [z(x,y) - z^{n - 1} (x,y)]\frac{{df[z(x,y)^{n - 1} ]}}{dz(x,y)} = 0$$

After iterative calculation, the depth $z^{n} (x,y)$ is obtained. On this basis, the relative concave–convex relationship in the image and the concave–convex relationship within each region are obtained through human interaction. Finally, the image depth information that meets the actual situation is obtained.

### Surface reconstruction

**Figure 6:**
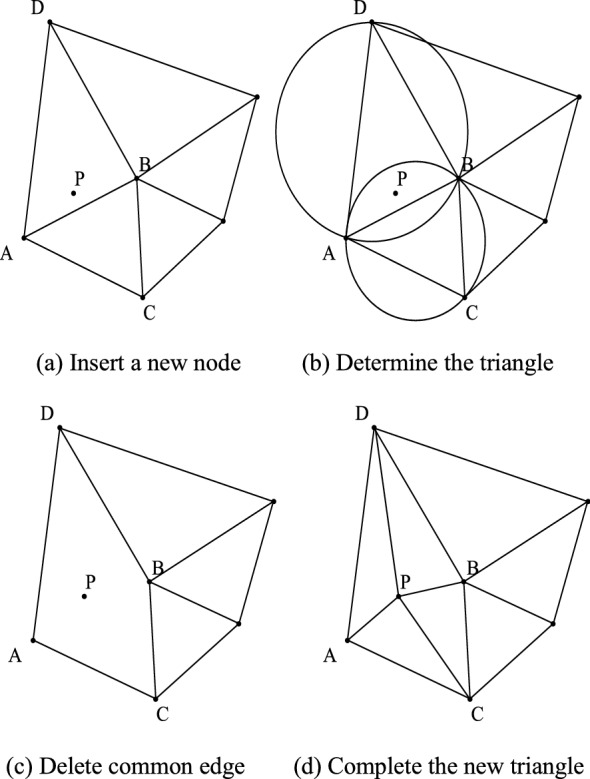
Steps to insert a point to construct a triangle link.

The point cloud data are optimized by the triangulation algorithm with the goal to obtain a symmetrical and efficient final surface. To achieve symmetry, researchers have combined the properties of triangulation and the optimal shape criterion, most of them using the Delaunay criterion^15–17^. These algorithms improve the calculation speed as much as possible while ensuring the uniform shape. In this paper, the Delaunay triangulation algorithm is used to triangulate the point cloud. The final bas-relief modeling effect is displayed by OpenGL. The basic steps are shown in Fig. 6.

1. A big triangle that can contain all points is constructed, and it is put in the triangle linked list.
2. The points for construction are inserted in turn. The circumcircle of the extended edge containing the insertion point is obtained while facing the insertion point; the common edge is deleted, and finally, the insertion point is connected with the vertices of the triangle in turn to complete the operation of an insertion point.
3. The optimization criteria are used to optimize the new triangle, which is then put into the triangle linked list.
4. Steps (2) and (3) are repeated to obtain the complete triangulation data.

## Experimental results and discussion

### Regional division results

**Figure 7:**
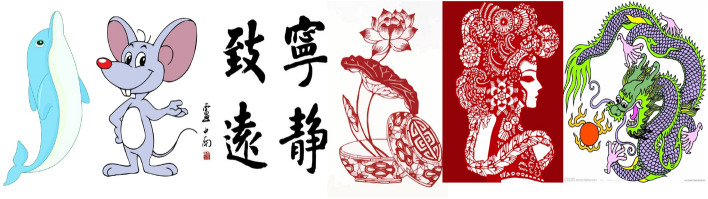
Original images.

**Figure 8:**
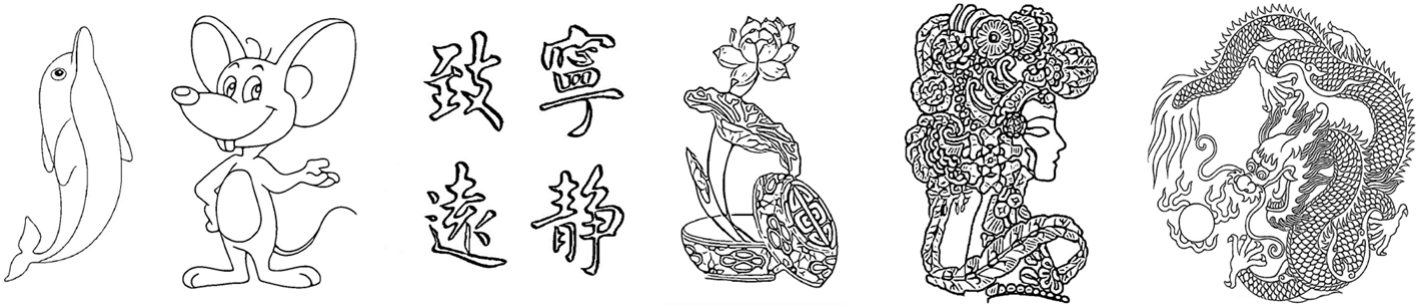
Images of contour extraction results.

**Figure 9:**
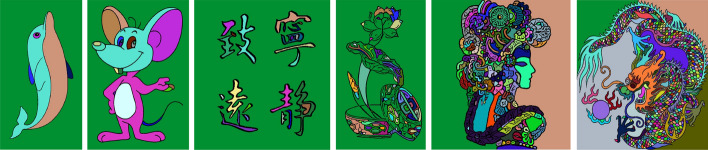
Images of regional division results.

This study conducted regional division tests on selected six groups of images. The original images and division results are shown in Figs. 7, 8 and 9.

Furthermore, the area division algorithm used in this study is compared with the traditional two-pass scanning method, seed filling method, and contour tracking method. The computer configuration of the tests is Dell OptiPlex 3020, i5 processor, and 4G memory. The operating environment is Win7 (64-bit), Visual Studio 2010, Opencv2.4.9, and Debug mode. The results are shown in Table 1.

**Table 1:** Performance comparison of different algorithms for different images (unit: s).

	Two-pass scanning method	Seed filling method	Traditional contour tracking method	Proposed method
1	1.23564	2.51726	3.41704	0.142681
2	1.16526	2.56931	3.25879	0.122032
3	2.82458	2.812546	4.921586	0.218534
4	3.135486	3.017265	5.486386	0.235847
5	1.23865	2.57824	3.51682	0.150389
6	2.614853	3.048513	5.501853	0.248513
Mean value	2.453392	2.864141	4.85666125	0.213320

Table 1 indicates that, compared with the traditional two-pass scanning method, seed filling method, and contour tracking method, the proposed regional division algorithm has less running time in image processing with different levels of complexity. Therefore, the performance is greatly improved.

### Deep recovery results

Image depth recovery can be judged by whether the hierarchical relationship between regions is obvious, and whether the concave–convex relationship between layers and that within each region are consistent with reality. Through the traditional SFS algorithm and the proposed algorithm, images with obvious hierarchical relationship in depth are recovered. The corresponding height information comparison images are obtained.

**Figure 10:**
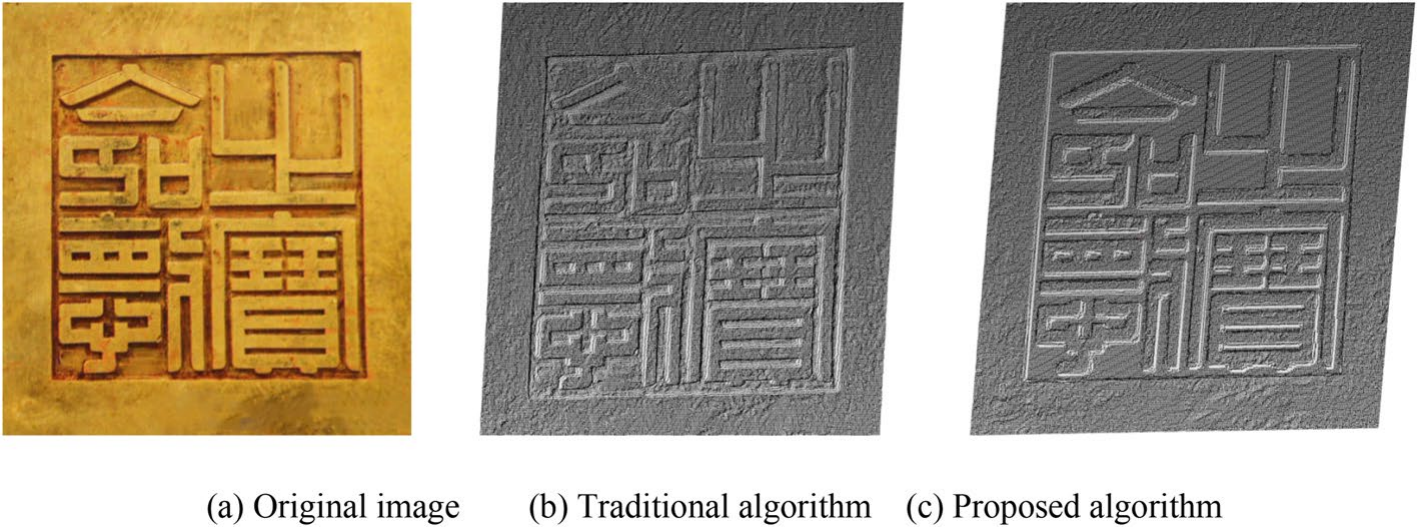
Point cloud comparison images of the “treasure seal”.

**Figure 11:**
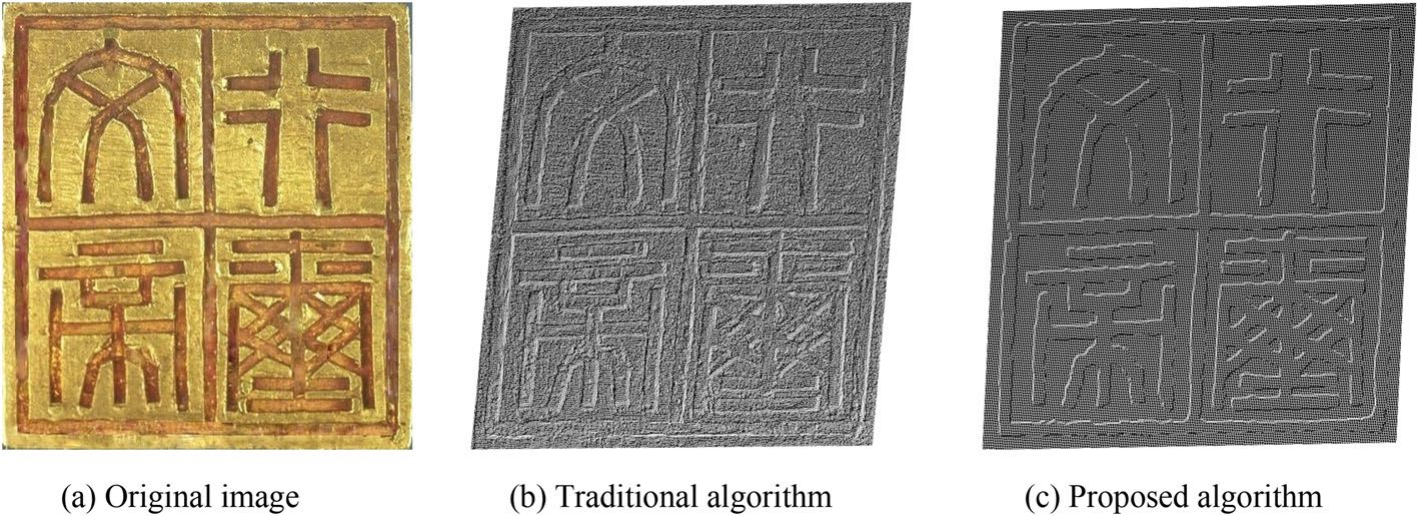
Point cloud comparison images of the “Wen Emperor seal”.

Figures 10 and 11 show the recovery effect of the height values of the “treasure seal” and “Wen Emperor seal”. In the height information recovered by the traditional algorithm, the height value of the text is lower or higher than that of the base surface. This is inconsistent with the height value information obtained by the human eye in the original image. Moreover, there is considerable noise effect and the surface is rough. The height information recovered by the proposed algorithm is basically the same as that judged in the original image. Furthermore, the noise points in the image are reduced, and the surface is relatively smooth.

**Figure 12:**
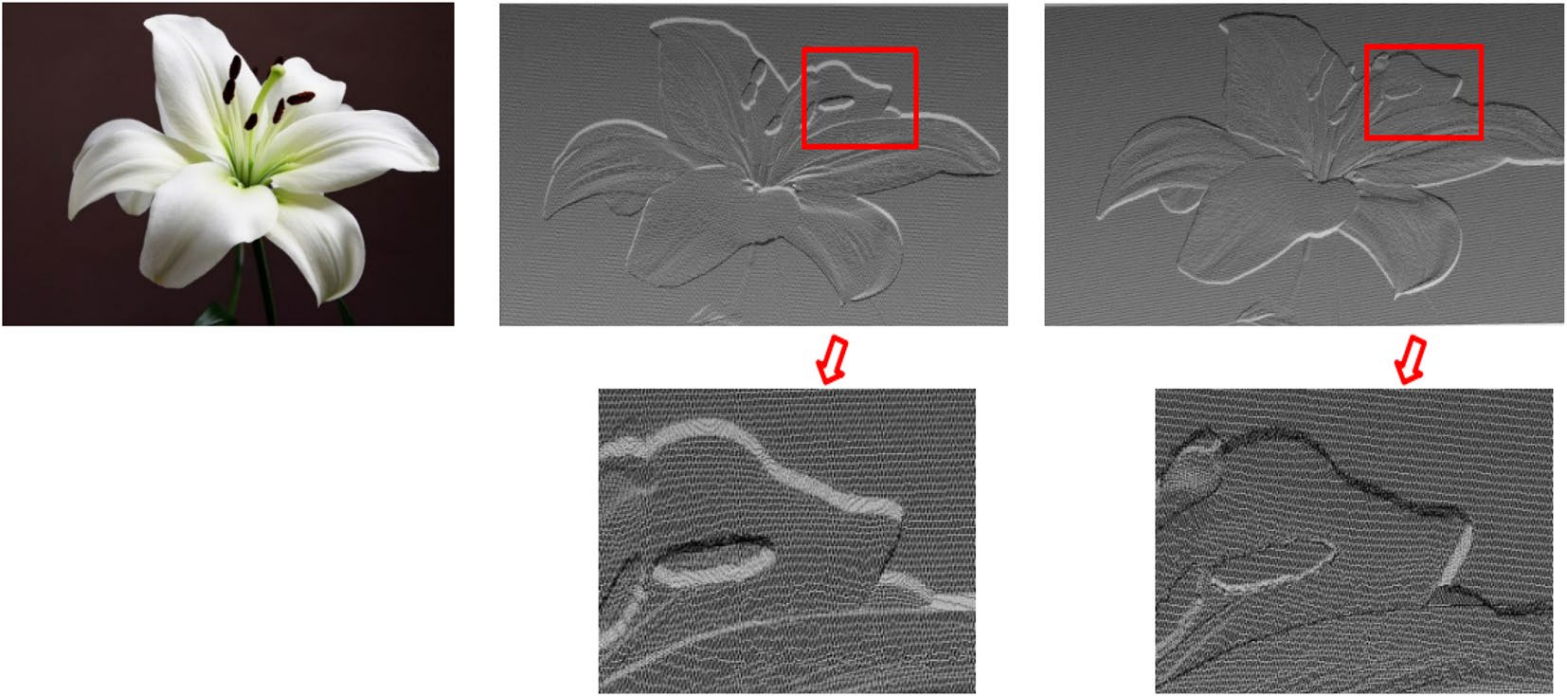
Point cloud comparison images of the “lily pattern”.

**Figure 13:**
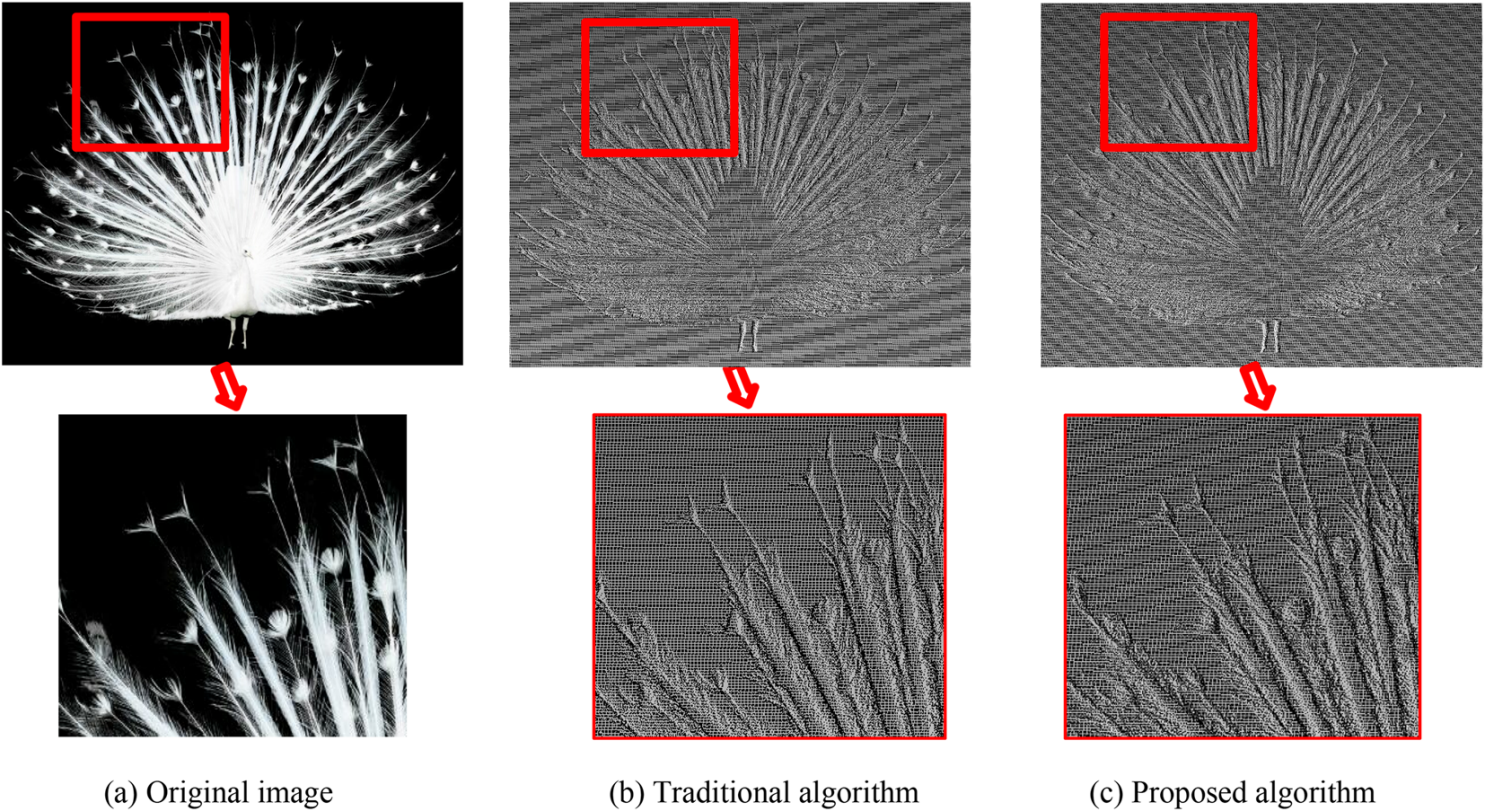
Point cloud comparison images of the “peacock pattern”.

Figure 12 shows the comparison of the depth recovery effect of the “lily pattern”. Both algorithms can recover the 3D contour of the image very well with less noise. In the image displayed by the point cloud, the light source is located at the upper right of the image. The image is observed from the upper right to the bottom of the image. In the traditional algorithm, the recovered depth of the lily in the image is generally lower than the plane where it is located, and the relative height of each region does not match the actual situation. The target depth recovered by the proposed algorithm is generally higher than the base surface where it is located. The relative depth between each region is consistent with the actual situation, and the effect of recovered details is better. Figure 13 shows the comparison of the height value recovery effect of the “peacock pattern”. In the height information obtained by the traditional algorithm, the overall height of the peacock is less than that of the plane where it is located, which does not constitute the bas-relief model we expect. The height of the peacock obtained by the proposed algorithm is higher than the plane where it is located, which is consistent with the actual situation. Moreover, the recovery effect is better in the details of the peacock feathers.

### 3D reconstruction results

**Figure 14:**
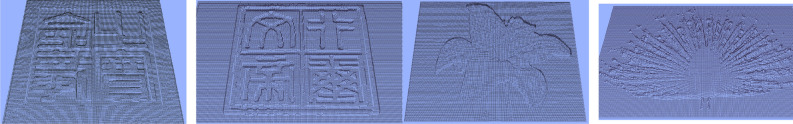
Relief renderings.

The point cloud data in Figs. 9, 10, 11, and 12 are reconstructed in three dimensions and displayed through OpenGL visualization. The reconstructed bas-relief models are shown in Fig. 14. Figures 9 and 10 show the bas-relief renderings of two different styles of seals. Their height information is obtained by the proposed algorithm. Through the triangulation algorithm, the surface of the finally generated bas-relief model is smoother, with obvious levels and accurate concave–convex relationship.

Figures 12 and 13 show the simple images of animals and plants. Because the relative height difference between each region is small and the details are more complicated, there is a certain degree of distortion in the later triangulation algorithm. The algorithm needs to be further optimized in the later stage.

**Figure 15:**
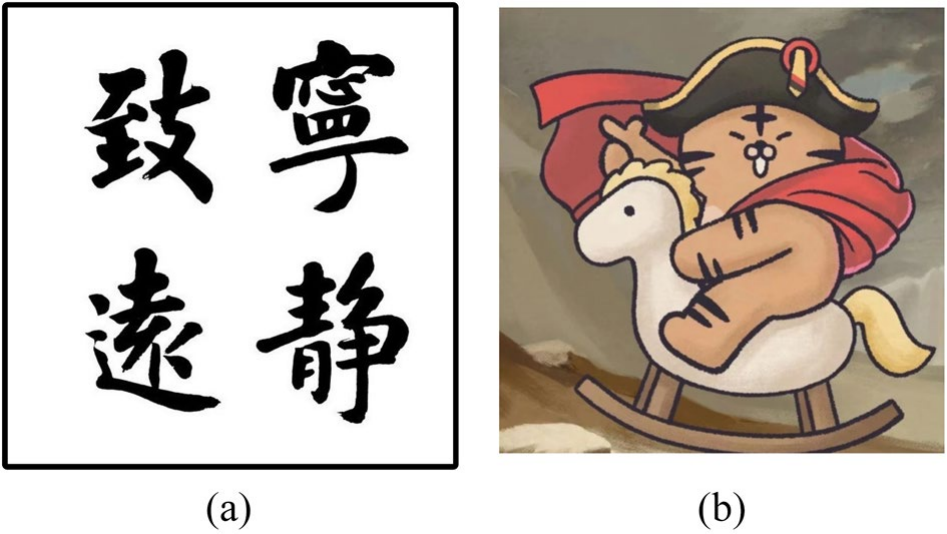
Original images: (**a**) the original images of simple calligraphy; (**b**) the original images of complex cartoon.

**Figure 16:**
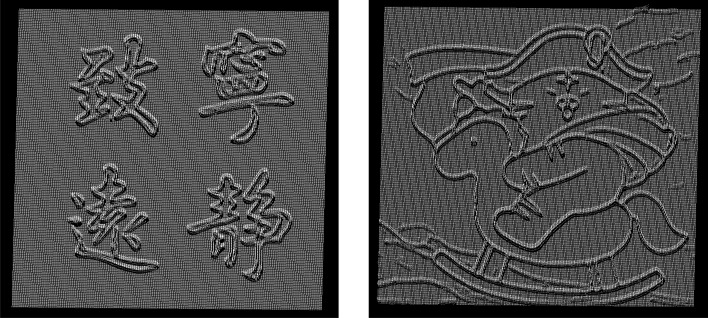
Image height value recovery point cloud.

In Figs. 15, the original images of simple calligraphy images with clear background and complex cartoon images with more contours, the relief renderings generated by the proposed algorithm are respectively shown in Fig. 16. Through the observation of the experimental results, it can be seen that no matter it is simple calligraphy or complex cartoon images, the algorithm in this paper makes the concave convex relationship between each area of the image obvious, and the effect of height restoration conforms to the height relationship seen by the human eye in the original image, which conforms to the actual situation, and the algorithm has strong anti noise interference ability, can achieve obvious and smooth contours.

**Figure 17:**
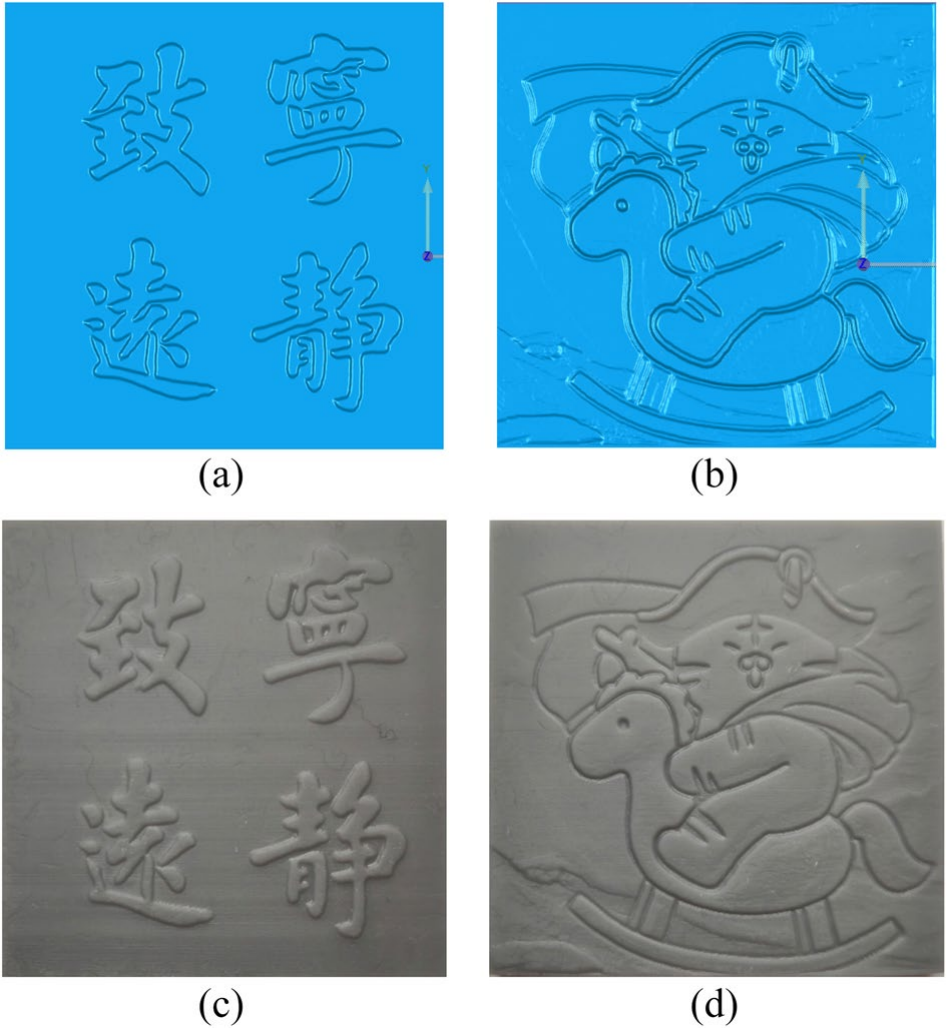
Image 3D reconstruction and 3D printing results: (**a**) the 3D reconstruction of simple calligraphy images; (**b**) the 3D reconstruction of complex cartoon images; (**c**) the 3D printing picture of simple calligraphy images; (**d**) the 3D printing picture of complex cartoon images.

Then, we make 3D reconstruction for image height value recovery point cloud in Fig. 16, and use the UV printer to 3D print the modeling results. The results are shown in Fig. 17a–d are the effect pictures of 3D printing.

Through the analysis of modeling results and 3D printed physical modeling, it can be seen that the algorithm in this paper has a good retention effect on high-frequency information of image edges, with obvious features, making the physical modeling more realistic, and the effect of height restoration conforms to the height relationship seen by the human eye in the original image. By observing the 3D reconstruction results of cartoon images, it can be found that the algorithm in this paper can distinguish the background and key information well, and the height changes smoothly, and the relief is clear without too many complex lines.

### Comparisons

This paper uses the analytic hierarchy process to evaluate the final bas-relief effect generated by the algorithm in this paper and the traditional SFS algorithm. We selected several groups of pictures (as shown in Figs. 10, 11, 12 and 13) for the experiment of shallow relief effect. The basic construction steps are:

#### Establishment of hierarchical structure model

The first layer is the target layer ($O$): this paper evaluates the effect of bas-relief modeling of digital images; The second layer is the criterion layer ($C$): determine the influencing factors for evaluating the effect of bas-relief. By testing 20 randomly selected people who participated in the effect evaluation experiment, 90% believed that the effect of bas-relief could be evaluated from four factors, surface smoothness, overall hierarchical relationship, concave convex relationship between regions, and actual compliance. The third layer is the scheme layer ($P$): $N(N \ge 2)$ evaluation objects, which are recorded as $P_{n} (n = 1,2)$.

#### Construct pairwise comparison matrix

The relative importance of criterion layer $k$ factors $C_{k} (k = 1,2,3,4)$ to the upper level $O$ measured on a scale of 1–9,$a_{ij} (i,j = 1,2, \ldots ,n)$.

The comparison matrix of evaluation indicators for the definition of bas-relief effect is shown in Table 2.

$${\text{Recorded as}}:A = \left[ {\begin{array}{*{20}c} 1 & 3 & 7 & 5 \\ {{1 \mathord{\left/ {\vphantom {1 3}} \right. \kern-\nulldelimiterspace} 3}} & 1 & 5 & 3 \\ {{1 \mathord{\left/ {\vphantom {1 7}} \right. \kern-\nulldelimiterspace} 7}} & {{1 \mathord{\left/ {\vphantom {1 5}} \right. \kern-\nulldelimiterspace} 5}} & 1 & 2 \\ {{1 \mathord{\left/ {\vphantom {1 5}} \right. \kern-\nulldelimiterspace} 5}} & {{1 \mathord{\left/ {\vphantom {1 3}} \right. \kern-\nulldelimiterspace} 3}} & {{1 \mathord{\left/ {\vphantom {1 2}} \right. \kern-\nulldelimiterspace} 2}} & 1 \\ \end{array} } \right].$$

**Table 2:** Comparison matrix of evaluation indicators for bas-relief effect.

Index	Surface smoothness	Overall hierarchical relationship	Concave convex relationship between regions	Actual compliance
Surface smoothness	1	3	7	5
Overall hierarchical relationship	1/3	1	5	3
concave convex relationship between regions	1/7	1/5	1	2
actual compliance	1/5	1/3	1/2	1

The maximum eigenvalue of $A$ is obtained $\lambda \max \approx 4.2195$, the corresponding normalized eigenvector is $$W_{1} = \left( {\begin{array}{*{20}c} {0.5646} & {0.2691} & {0.0895} & {0.0768} \\ \end{array} } \right)^{T}$$.

#### Computational weight vector and its consistency test

In the actual construction of comparison matrix, it is impossible to satisfy the condition that it is completely consistent,the formula is satisfied $a_{ij} a_{jk} = a_{ik} ,1 \le i,j,k \le n$, therefore, we only need to find that certain consistency of the matrix.

$$CI = \frac{{\lambda_{\max } - n}}{n - 1} \approx \frac{4.2195 - 4}{{4 - 1}} \approx 0.0732$$

The size of $CI$ indicates the consistency of the comparison matrix. In order to measure the consistency of comparative evidence, a random consistency index $RI$ is introduced. The data of this index is obtained from experience, as shown in Table 3:

**Table 3:** Standard value of average random consistency index.

Matrix order	1	2	3	4	5	6	7	8	9	10
RI	0	0	0.58	0.90	1.12	1.24	1.32	1.41	1.45	1.49

In this paper, the matrix order for $A$ is 4, so take $RI = 0.90$.the rate of $CI$ and $RI$ is called consistency ratio indicator,expressed as $CR$, when $CR < 0.10$, the consistency of the matrix meets the requirements, otherwise, the proportion of influencing factors shall be taken again. It is calculated that:

$$CR = \frac{CI}{{RI}} = \frac{0.0732}{{0.90}} \approx 0.0813 < 0.1$$

Here $CR < 0.10$, the evaluation index comparison matrix in this paper meets the conditions,$W_{1}$ take as the weight vector $C$ layer to $O$ layer.

Twenty randomly selected people compared the effect of the algorithm in this paper with that of the traditional SFS algorithm. The score was made from four aspects: whether the surface was smooth, whether the hierarchical relationship between regions was obvious, whether the concave convex relationship between regions was correct, and whether the image relief effect was consistent with the visual effect. The scoring criteria were: the score was 1–5, and the corresponding effect was from weak to strong. Take the average scores of 20 people, and get the quantitative scores in Table 4.

**Table 4:** Quantitative score table.

Group	Surface smoothness	Overall hierarchical relationship	Concave convex relationship between regions	Actual compliance
Traditional algorithm	3.00	3.45	2.65	2.7
Algorithm in this paper	3.85	3.65	3.95	3.60

According to Table 4, the comparison matrix between the construction layer $P$ and the criterion layer $C_{k}$ can be obtained:

$$B_{1} = \left[ {\begin{array}{*{20}c} 1 & {\frac{3.00}{{3.85}}} \\ {\frac{3.85}{{3.00}}} & 1 \\ \end{array} } \right]B_{2} = \left[ {\begin{array}{*{20}c} 1 & {\frac{3.45}{{3.65}}} \\ {\frac{3.65}{{3.45}}} & 1 \\ \end{array} } \right]B_{3} = \left[ {\begin{array}{*{20}c} 1 & {\frac{2.65}{{3.95}}} \\ {\frac{3.95}{{2.65}}} & 1 \\ \end{array} } \right]B_{4} = \left[ {\begin{array}{*{20}c} 1 & {\frac{2.70}{{3.60}}} \\ {\frac{3.60}{{2.70}}} & 1 \\ \end{array} } \right]$$

After calculation, the maximum eigenvalues of $B_{1}$, $B_{2}$, $B_{3}$, $B_{4}$ is $\lambda_{\max }^{{}} = 2$, after normalizing the eigenvector, the weight vector is obtained:

$$W_{2} = \;\left( {\begin{array}{*{20}c} {\begin{array}{*{20}c} {0.4379} & {0.4862} & {0.4015} & {0.4286} \\ \end{array} } \\ {\begin{array}{*{20}c} {0.5621} & {0.5138} & {0.5985} & {0.5714} \\ \end{array} } \\ \end{array} } \right)$$

Then the weight of $P$ to $O$ is:

$$W = W_{2} \cdot W_{1} = \left( {\begin{array}{*{20}c} {0.4469} \\ {0.5531} \\ \end{array} } \right)$$

According to the ranking of comprehensive strength indicators from large to small, the algorithm in this paper is better than the traditional algorithm.

## Conclusion and future work

In this study, RGB monocular images were adopted as the research object and a method of bas-relief modeling for RGB monocular images based on regional division was proposed. First, the edge contour of the RGB monocular image was extracted and refined by the Gaussian difference algorithm of the tangential flow. Then, regional division was performed on the RGB monocular image using the improved connected-domain labeling algorithm. Subsequently, the SFS algorithm and human–computer interaction were used to generate a 3D point cloud of each image region that meets the concave–convex characteristics of the bas-relief relief. Finally, the bas-relief model of the RGB monocular image was obtained by reconstructing the triangular facets. This method can automatically obtain the pixel height value of each image region in the image and adjust the concave–convex relationship of each image region to realize a bas-relief model based on the RGB monocular image.

Our future work will focus on the more complex images. In the process of using the extracted image contour to perform regional division, a slight difference exists between the obtained result and the actual situation at the contour; this affects the later image depth value recovery to a certain extent. Furthermore, distortion may occur in the process of 3D reconstruction, which requires further research on the processing of point cloud data to reconstruct a high-quality 3D surface.
